# Large-Scale and Flexible Self-Powered Triboelectric Tactile Sensing Array for Sensitive Robot Skin

**DOI:** 10.3390/polym9110586

**Published:** 2017-11-07

**Authors:** Huicong Liu, Zhangping Ji, Hui Xu, Ming Sun, Tao Chen, Lining Sun, Guodong Chen, Zhenhua Wang

**Affiliations:** School of Mechanical and Electric Engineering, Jiangsu Provincial Key Laboratory of Advanced Robotics, Collaborative Innovation Center of Suzhou Nano Science and Technology, Soochow University, Suzhou 215123, China; hcliu078@suda.edu.cn (H.L.); jizhangping@foxmail.com (Z.J.); yanwu19@126.com (H.X.); sunming08@126.com (M.S.); lnsun@hit.edu.cn (L.S.); guodongxyz@163.com (G.C.); wzh@hit.edu.cn (Z.W.)

**Keywords:** flexible electronics, triboelectric tactile sensing array, robot skin, robotic tactile feedback system

## Abstract

Advances in flexible and multifunctional electronic devices have enabled the realization of sophisticated skin for robotics applications. In this paper, a large-scale, flexible and self-powered tactile sensing array (TSA) for sensitive robot skin is demonstrated based on the triboelectric effect. The device, with 4 × 4 sensing units, was composed of a top triboelectric polyethylene terephthalate (PET) layer, a bottom triboelectric copper (Cu) layer and a bottom PET substrate. A low-cost roll-to-roll ultraviolet embossing fabrication process was induced to pattern the large-scale top PET film with microstructures for high-output performance. The working mechanism and output performance of the triboelectric TSA were demonstrated and characterized, exhibiting good stability and high sensitivity. By integrating a tactile feedback system, the large-scale TSA, acting as intelligent skin for an industrial robot, was able to realize emergency avoidance and safety stop for various unknown obstacles under various working conditions. The system also has good real-time performance. By using a large-scale roll-to-roll fabrication method, this work pushes forward a significant step to self-powered triboelectric TSA and its potential applications in intelligent robot skin.

## 1. Introduction

Tactile sensing is a key technology for intelligent robotics able to operate in unstructured environments and conduct safe interactions with humans and objects. A flexible robot skin capable of tactile sensing over a large area is expected to broaden the cognitive capability of robots, and to enhance the autonomous movement capability within an unknown environment. In comparison to tactile sensors, non-contact sensors are more susceptible to the ambient environment. Owing to harsh environments and complex industrial requirements, non-contact sensors are easily confused, and make poor decisions, which could lead to a hidden risk of accidents. Flexible tactile sensors have been widely investigated in the past few decades, and many sensing mechanisms have been reported in the literature, including capacitive [1,2,3,4], inductive, resistive [5,6,7,8], piezoelectric [9,10,11], thermoelectric [12], and other functional materials with sensing characteristics [13,14,15,16]. However, the complicated fabrication process and low scalability of these methods still remain critical obstacles to the mass production of large-scale robot skin. Moreover, most of these sensors rely on an external power supply to work, thus potentially making the electrical wiring of a large number of sensor arrays on existing industrial robots more complicated.

In this paper, we introduce a high-performance self-powered TSA based on a triboelectric mechanism. The contact and separation process between two triboelectric layers introduces surface charge transfer, i.e., conversion of the external mechanical force into an electrical output signal without an external power source. It also has the advantages of easy fabrication, high efficiency and a wider choice of materials. To achieve high sensitivity of the TSA, the surface morphology of the triboelectric contact layers is crucial for charge generation in the process of contact electrification [17,18,19]. Therefore, domestic and foreign researchers have figured out different methods to improve the surface morphology and effectiveness, such as block copolymer self-assembly [20,21], soft lithography [22,23], nanoparticle deposition [24], chemical treatment [25,26,27] and plasma etching [28,29,30]. However, these methods are critically limited by the chamber or wafer size, and hence can only fabricate relatively small-sized samples at high cost. They are not beneficial for mass production and commercialization of flexible triboelectric TSA. We demonstrated a process flow of roll-to-roll UV embossing for patterning large-sized PET film with mass replicate microstructures. The mass surface treatment of the PET friction material could effectively enhance the triboelectrification effect of the TSA [31].

The flexible TSA as artificial electronic skin is expected to accommodate irregular surfaces, and imitate the human somatosensory system for potential applications in health monitoring, artificial prosthetics and advanced robotics [32]. Mei et al. applied their flexible tactile sensor on a prosthetic hand for measuring the grasping force [33]. They designed a typical signal-processing circuit for the application, including a microcontroller unit (MCU) and an analog digital conversion unit [34,35,36]. In this work, we successfully demonstrated a tactile feedback system targeting industrial robot, in which the signal-processing circuit worked as the interface connecting the TSA to the robot controller. The tactile feedback system has good real-time performance, and the emergency avoidance and stop functions were implemented efficiently at the minimum impacting velocity of 0.12 m/s.

## 2. Materials and Methods

**Figure 1:**
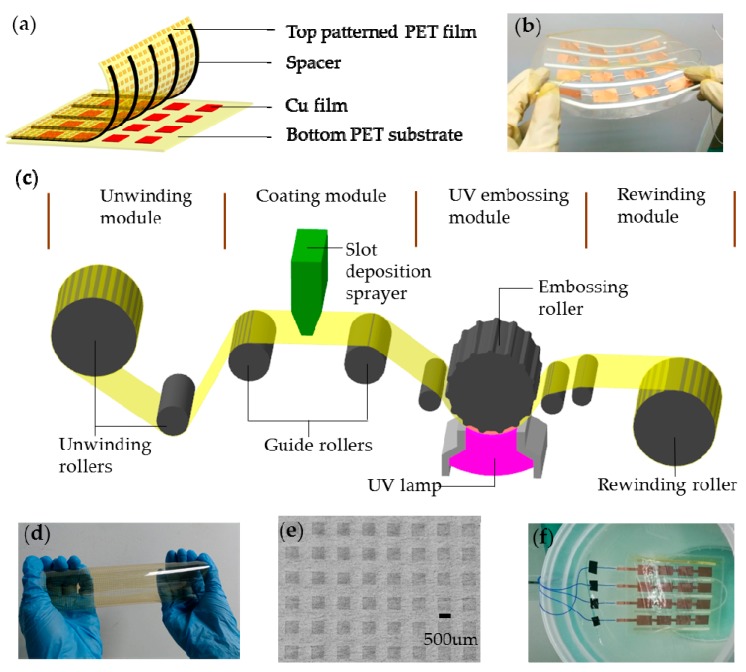
(**a**) Schematic diagram of the flexible TSA with micro patterns on top PET film; (**b**) Photograph of the flexible TSA sheet; (**c**) Schematic illustration of roll-to-roll UV embossing fabrication setup; (**d**) Photograph of the top patterned PET film; (**e**) Optical image of the microstructures on patterned film; (**f**) Photograph of the TSA after sealing and waterproofing treatment.

A schematic diagram of the flexible triboelectric TSA is illustrated in Figure 1a. It is composed of a top-patterned PET film of 125-μm in thickness acting as the top triboelectric layer, and a 30-μm-thick Cu film attached on a 125-μm-thick bottom PET substrate used as the bottom triboelectric layer. 1-mm-thick foam tape was attached on the top-patterned PET film acting as spacer. The 4 × 4 Cu film units of 3 cm × 2 cm in area size were laminated onto the bottom PET substrate. The spacer was conductive, to realize the contact and separation process between the large patterned PET film and each Cu unit when it was subjected to mechanical force. The top and bottom components were then integrated together to form a flexible TSA, as shown in Figure 1b. It can be seen that the TSA has good flexibility and can be easily bent by hand.

For the fabrication of the large-scale patterned PET triboelectric layer, roll-to-roll UV embossing was implemented, as illustrated in Figure 1c. It can be divided into four functional modules, which are: (1) unwinding module for providing blank PET substrate; (2) coating module for supplying UV curable resin deposited on PET substrate; (3) UV embossing module designed to pattern microstructures on PET substrate; and (4) rewinding module for generating web tension to separate the embossed PET film from the embossing roller and collecting the embossed PET film into a roller. In fabrication process, firstly, the PET film was pre-coated with a primer layer composed of a functionalized α-olefin made up of copolymers and cross-linking agents, which was used to increase the adhesiveness of the coated UV-sensitive resin layer in the roll-to-roll UV embossing process. Then a 125-μm-thick PET film was coated with UV-curable resin through a slot die. Afterwards, a patterned roller was adopted in the UV embossing module for transferring the micro patterns onto the coated PET film, and a UV lamp installed beneath the embossing roller was used to cure the transferred microstructures onto the PET film. After coating, embossing and separation steps, micro patterns on the patterned roller were finally transferred onto PET film. The large-scale top PET film was patterned with microstructures via roll-to-roll UV embossing method and then cut into size sheet of 20 cm × 20 cm. The microstructures on PET sheet were square pillars of 500 μm in length and 25 μm in height, which were used to increase the performance of the triboelectric effect based on contact electrification. Figure 1d shows the photograph of the fabricated PET film sheet with microstructures, and Figure 1e is the enlarged optical image of the patterned microstructures of the pillar array.

The flexible TSA was designed as sensitive skin for an industrial robot, for tasks such as sorting and conveying robots, polishing and painting robots, and so on. In the harsh industrial environment, the device is easily contaminated by dust, particles, paints, vapor and so on. For the sake of good and stable output performance, sealing and waterproofing treatment around the sheet was conducted by using polydimethylsiloxane (PDMS). We chose PDMS as sealing material due to its characteristics of good flexibility and leakproofness. A 200-μm-thick PDMS (Sylgard 184 base: curing agent = 10:1 in weight) was smeared onto each edge of the TSA, covering each gap. After curing the PDMS at 50 °C for 4 h and removing the surrounding excess PDMS, the sealing treatment for the TSA was completed. After the sealing treatment, the large-scale TSA was immersed into water as shown in Figure 1f and it was verified that all wire connections were working well.

## 3. Results and Discussion

### 3.1. Characterization

**Figure 2:**
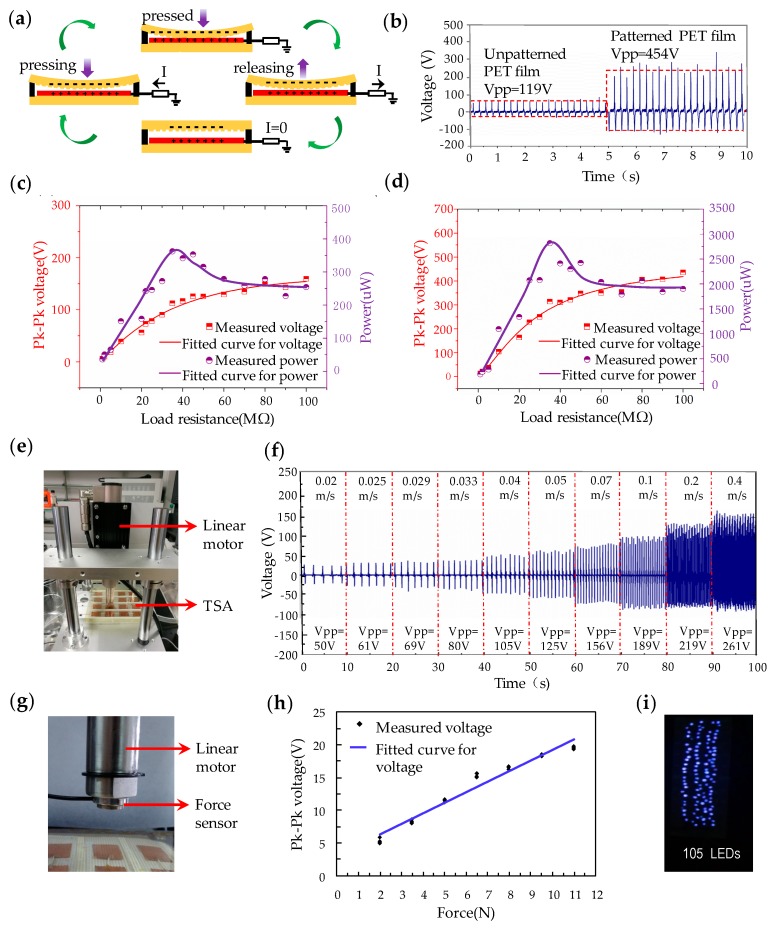
(**a**) Operating mechanism of the triboelectric TSA; (**b**) Output voltage of the TSA by using unpatterned and patterned PET; Voltage and power transferred to different load resistors of the TSA by using (**c**) unpatterned and (**d**) patterned PET; (**e**) Measurement setup for studying the sensing performance of the TSA; (**f**) Output voltage generated by the TSA at different speeds of the external force; (**g**) Experimental setup to measure the pressure applied on the TSA; (**h**) Peak-to-peak voltage generated by the TSA at different applied pressures; (**i**) Each patterned triboelectric sensing unit can simultaneously light up 105 LEDs by finger tapping.

Figure 2a shows the operating mechanism of the triboelectric TSA by the coupling of contact electrification and electrostatic induction. In the non-contact state, the patterned top PET film is separated from the bottom Cu film by a spacer, and both layers are uncharged. When an external mechanical force is applied, such as finger tapping, both the triboelectric layers are brought into full contact. According to the triboelectric series, electrons are transferred onto the patterned PET film from the Cu film because the PET tends to be more triboelectrically negative than Cu in the contact electrification process. The generated triboelectric charges with opposite polarities then approach balance, leading to there being no current in the external circuit. When the applied force is released, the patterned PET film and Cu film start to separate, leading to electric potential difference, which generates current flowing from the Cu film to the reference ground. When these two films separate to the maximum position, there will be no current through the load resistor connected between the Cu film and the ground. When the external force is applied again, the PET film begins approaching the Cu film. This causes a reverse-orientation electric current in the load resistor, until the patterned PET film and Cu films are fully in contact with each other again. This is a working cycle of electricity generation in contact-separation mode due to external force applied on the TSA.

To study the effect of embossed patterns on the performance of triboelectric mechanism, both the fabricated TSA with and without embossed patterns were characterized. Figure 2b shows the output voltages of the triboelectric TSA by finger tapping using unpatterned and patterned top PET triboelectric layers. The corresponding peak-to-peak voltages were measured to be 119 and 454 V, respectively. It can be seen that the embossed patterns of the PET film led to improved triboelectric performance due to easier charge separation, as demonstrated by Fan et al. [19]. Figure 2c,d illustrates the variations of voltage and power transferred to different load resistors for the TSA with unpatterned and patterned microstructures, respectively. The measured peak-to-peak voltage increased continuously as the value of the connected load resistance increased. The maximum power of the TSA with patterned microstructures was obtained to be 2.82 mW at a matched load resistance of 35 MΩ, corresponding to an output power density of 0.47 mW/cm^2^, which was much higher than the values for the TSA without microstructure patterns.

In the dynamic sensing process, the speed and frequency of the applied force may constantly change, resulting in variable output performance of the triboelectric TSA. The dynamic characterization was conducted by a linear motor system (Linmot E1100, NTI AG, Spreitenbach, Switzerland), consisting of a slider, a stator and a motion controller, as shown in Figure 2e. The end effector of the linear motor is able to be accurately positioned at any point of the full-stroke at various speeds. A force sensor (LSZ-F08, SZOBTE, Suzhou city, China) was fixed to the bottom of the slide bar of the linear motor to measure the force applied to the TSA, as shown in Figure 2g. The TSA of 4 × 4 sensing units was placed under the linear motor, and each unit was applied a fixed dynamic force with different speeds ranging from 0.02 m/s to 0.4 m/s. Figure 2f shows the variation of the peak-to-peak voltages of the TSA under different speeds of the external force. It is fairly obvious that the output performance of the TSA correlates well with the speed of the applied probe. As the probe speed was increased to 0.02, 0.05, 0.1, 0.2 and 0.4 m/s, the corresponding peak-to-peak voltage increased to 50, 125, 189, 219 and 261 V, respectively. The output increment is caused by faster electron flow, while the charge transfer is maintained under higher dynamic probe speed [37]. In the next, the experimental setup shown in Figure 2g was utilized to measure the output voltage of the TSA against the amplitude of the applied force. It can be observed from Figure 2h that the output voltage increases linearly as the force applied to the sensing unit increases. The force sensitivity of the sensing unit was characterized to be 1.14 V kPa^−1^. The output increment can be attributed to the increment of contact area due to larger contact force [38]. Figure 2i demonstrates that each patterned triboelectric unit of the TSA was able to simultaneously light up 105 LEDs by finger tapping.

### 3.2. Tactile Feedback System

The fabricated large-scale TSA acting as flexible electronic skin has wide application prospects in advanced robotics. For instance, industrial robots with multiple degrees of freedom can realize complex working procedures driven by servo system. Because industrial robots cannot recognize the unknown or unexpected obstacles, there is a huge security risk in the production line. Although there are strict safety procedures and protective fences on site, robot injury accidents occur frequently due to erroneous operation or unexpected intrusion of people or objects. In order to avoid such accidents, a tactile feedback system integrating flexible TSA on a robotic arm was developed for use in an obstacle-avoidance safety application. When an unexpected intrusion of people or objects runs into an arbitrary sensing unit of the TSA, the working robot should be able to detect the tactile force and respond quickly to avoid adverse consequences.

**Figure 3:**
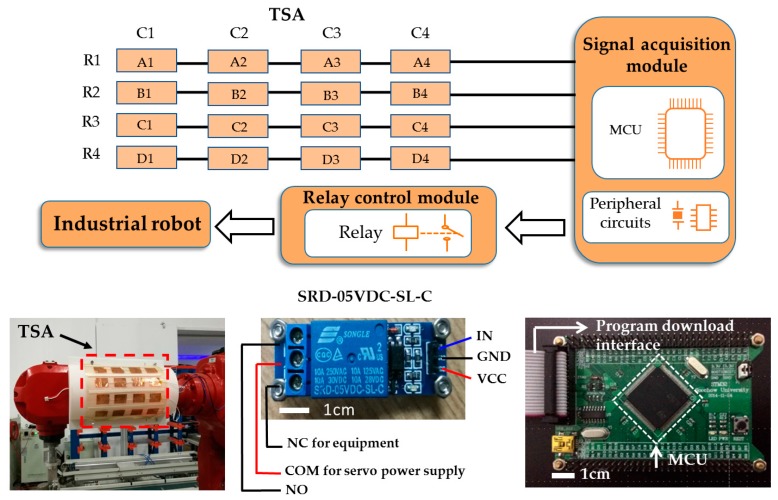
Schematic diagram of a tactile feedback system for an industrial robot, and photographs of the industrial robot, relay control module PCB and the signal-acquisition module PCB.

Figure 3 illustrates the diagram of the tactile feedback system. It consists of four parts: self-powered TSA, signal acquisition module, relay control module and industrial robot. The flexible TSA of 4 × 4 units was attached on the robotic arm in order for precise self-sensing of external tactile force to be possible. The output voltage of any triboelectric sensing unit can be detected by the signal acquisition module, and an interrupt program can be triggered accordingly. The interrupt command enables the relay control module to open its switch and cut off the servo power supply, resulting in emergency stop of the industrial robot.

The signal acquisition module is mainly comprised of a MCU and a peripheral circuit. The MCU is employed for data acquiring, processing, sending, and program commands processing. The relay control module is composed of an electromagnetic relay (SRD-05VDC-SL-C, Songle, Ningbo city, China) and some auxiliary electronic components. When an external force is applied to the triboelectric sensing unit, which indicates that the industrial robot has encountered an unknown obstacle during movement, the output voltage of the TSA is transmitted to the MCU, triggering the interrupt program. While executing the interrupt service program, a high-level signal is sent to the relay control module. Thereafter, the interrupt program is completed, and the main program continued until the signal-acquisition module stops running. The output terminal of the relay control module is connected to the controller of the industrial robot so as to turn the servo power supply on/off.

### 3.3. Application on Industrial Robot

The tactile feedback system was successfully demonstrated on an industrial robot from Jiangsu Huibo Robot Co. Ltd, which was composed of base, arm, forearm, joints and electrical cables, and can realize multi-degree-of-freedom movement. The overall height of the arm and the base was 157 cm and the length of the forearm was 89 cm. The integrated tactile feedback circuit modules were encapsulated in a curved plate whose surface was covered by the TSA. The whole tactile feedback system was fixed on the forearm of the industrial robot to carry out the experiments. According to the robot safety operation specifications, industrial robots should include emergency stop control circuits. The emergency stop circuit is used to switch on and off the power supply of the servo driver. Once the power supply of the servo driver is cut off, the braking device of the servo motor starts to work, and the robot stops. The emergency stop control circuit was powered by 24 V DC power supply. The COM terminal of the relay control module was connected to the 24 V DC power supply, while the NC terminal was connected to the emergency stop circuit. When the COM terminal is changed to NO terminal, the power supply for servo driver is cut off, and the robot runs to emergency stop. 

**Figure 4:**
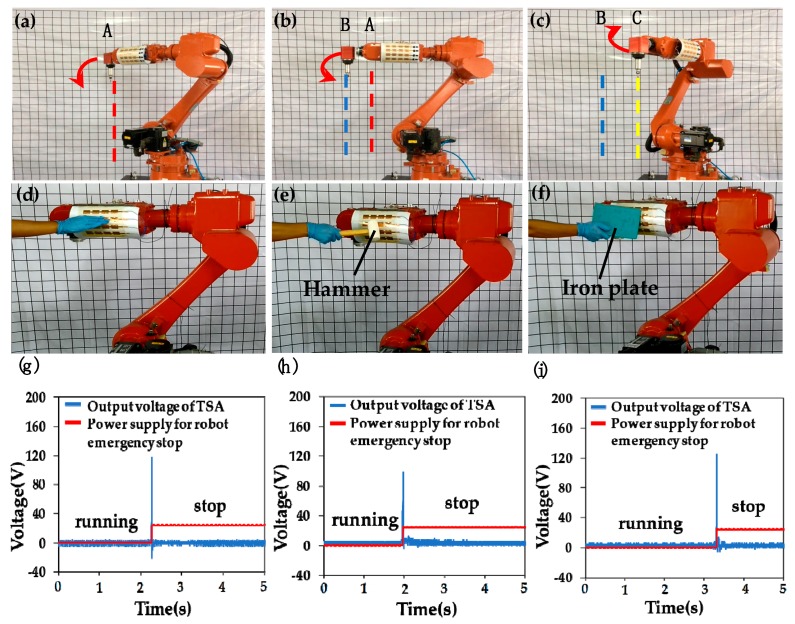
Experimental results of the tactile feedback system for application of robot obstacle avoidance; (**a**–**c**) The movement of the forearm without interruption; (**d**–**f**) Obstacles like hand, hammer and metal plate applied on the TSA during the movement of the forearm; (**g**–**i**) Real-time performance of the tactile feedback system.

Based on the prescribed running procedure, the robot forearm repeated a horizontal swing, i.e., moving forward from position A, to B, to C, and then backward from position C, to B, to A, as illustrated in Figure 4a–c. The red arrow represents the direction of movement. Once an unexpected obstacle suddenly appears in the movement path of the robot forearm, the obstacle encounters the sensitive TSA. Consequently, the output voltage of the sensing unit triggers the interrupt program. Hence, the signal-acquisition module controls the relay to cut off the servo power supply. Thus, the robot achieves emergency stop, and avoids further adverse consequences. In Figure 4d–f, we carried out a series of tactile feedback experiments by using different obstacles, such as a human hand, a rubber hammer and a metal plate. It was found that the TSA was very sensitive for immediately detecting various objects and feedback under unknown circumstances. (Videos S1, S2 and S3 show the tactile feedback experiments of the industrial robot with sensitive TSA by using different obstacles, being a human hand, rubber hammer and metal plate, respectively. The implementation of the emergency stop can be observed clearly.) To analyze the real-time response of the tactile feedback system, the output signal of the sensing array and the corresponding on/off states of the power supply for the robot emergency stop were recorded when the TSA encountered different obstacles as shown in Figure 4g–i. The pulse signal of the TSA indicated that it had encountered an obstacle. The sudden increase in voltage of the emergency stop circuit represented a sudden change in the movement state of the robot, i.e., from running to stopped. From these figures, it can be concluded that when the running robot experiences an unexpected force from different obstacles at any point in time, the tactile feedback system will respond quickly. The tactile feedback system has good real-time performance.

To measure the performance of the tactile feedback system, the emergency stop experiment of the robot forearm was conducted at different running speeds, which means that the TSA approached the obstacles at different dynamic forces. After repeated testing, the TSA was seen to be sensitive and effective between a minimum speed of 0.12 m/s and a maximum speed of 2.37 m/s relative to an obstacle. The TSA as a sensitive robot skin maintains high detection capability at a running speed of 0.12 m/s, which proves the tactile feedback system using the TSA is able to realize an emergency stop function even at very low impact speeds. (Videos S4, S5 and S6 show the tactile feedback experiments of the industrial robot with the sensitive TSA by using a human hand at different running speeds, which are low speed, medium speed and high speed, respectively. The implementation of emergency stop can be observed clearly even at extremely low running speeds.)

## 4. Conclusions

A flexible large-scale TSA based on the triboelectric effect was designed and characterized in this paper. It is able to transform the applied force into voltage signal without an external power supply. Moreover, a low-cost roll-to-roll embossing fabrication method was utilized to realize mass surface treatment of the PET film, and to enhance the sensitivity of the TSA. The tactile sensing characteristics indicated that the microstructured patterns on PET film fabricated by large-scale roll-to-roll embossing process contributed to the excellent output performance of the TSA. We also demonstrated a tactile feedback system for the application of emergency avoidance and safety stop towards industrial robots. The flexible self-powered TSA showed high detection capability and fast response in an unconstructed environment, and would be of benefit for the future secure interactions of humans and robots.

## Supplementary Materials

The following are available online at www.mdpi.com/2073-4360/9/11/586/s1, Video S1: tactile feedback experiment by using human hand, Video S2: tactile feedback experiment by using a rubber hammer, Video S3: tactile feedback experiment by using a metal plate, Video S4: tactile feedback experiment of the industrial robot at low speed, Video S5: tactile feedback experiment of the industrial robot at medium speed, Video S6: tactile feedback experiment of the industrial robot at high speed.
